# Comprehensive Profiling of Tubby-Like Proteins in Soybean and Roles of the *GmTLP8* Gene in Abiotic Stress Responses

**DOI:** 10.3389/fpls.2022.844545

**Published:** 2022-04-25

**Authors:** Hong-Ru Xu, Ying Liu, Tai-Fei Yu, Ze-Hao Hou, Jia-Cheng Zheng, Jun Chen, Yong-Bin Zhou, Ming Chen, Jin-Dong Fu, You-Zhi Ma, Wen-Liang Wei, Zhao-Shi Xu

**Affiliations:** ^1^College of Agriculture, Yangtze University/Hubei Collaborative Innovation Center for Grain Industry/Engineering Research Center of Ecology and Agricultural Use of Wetland, Ministry of Education, Jingzhou, China; ^2^Institute of Crop Science, Chinese Academy of Agricultural Sciences (CAAS)/National Key Facility for Crop Gene Resources and Genetic Improvement, Key Laboratory of Biology and Genetic Improvement of Triticeae Crops, Ministry of Agriculture, Beijing, China; ^3^College of Agronomy, Anhui Science and Technology University, Fengyang, China

**Keywords:** tubby-like protein, genome-wide analysis, abiotic stress, responsive mechanism, soybean

## Abstract

Tubby-like proteins (TLPs) are transcription factors that are widely present in eukaryotes and generally participate in growth and developmental processes. Using genome databases, a total of 22 putative *TLP* genes were identified in the soybean genome, and unevenly distributed across 13 chromosomes. Phylogenetic analysis demonstrated that the predicted GmTLP proteins were divided into five groups (I-V). Gene structure, protein motifs, and conserved domains were analyzed to identify differences and common features among the GmTLPs. A three-dimensional protein model was built to show the typical structure of TLPs. Analysis of publicly available gene expression data showed that *GmTLP* genes were differentially expressed in response to abiotic stresses. Based on those data, *GmTLP8* was selected to further explore the role of TLPs in soybean drought and salt stress responses. *GmTLP8* overexpressors had improved tolerance to drought and salt stresses, whereas the opposite was true of *GmTLP8*-RNAi lines. 3,3-diaminobenzidine and nitro blue tetrazolium staining and physiological indexes also showed that overexpression of *GmTLP8* enhanced the tolerance of soybean to drought and salt stresses; in addition, downstream stress-responsive genes were upregulated in response to drought and salt stresses. This study provides new insights into the function of GmTLPs in response to abiotic stresses.

## Introduction

The Tubby-like proteins (TLPs) are a class of eukaryotic transcription factors that were originally identified in obese mice (Kleyn et al., 1996; Liu, 2008). A typical TLP has a highly conserved tubular domain composed of 270 amino acids at the C-terminal, forming a β-barrel with a central hydrophobic α-helix and 12 antiparallel strands. It binds specific phosphatidylinositol 4,5-diphosphates to properly connect to the plasma membrane (Santagata et al., 2001; Mukhopadhyay and Jackson, 2011). TLPs have been widely studied in animals. For example, TULP3 is defined as a universal adapter for the transport of integral membrane proteins in the ciliary membrane to the cilia (Badgandi et al., 2017). Mutations of *TLPs* in humans lead to delayed obesity (Coleman and Eicher, 1990; Kleyn et al., 1996; Noben-Trauth et al., 1996; Kapeller et al., 1999; Borman et al., 2014), and mice with *TLP* mutations develop retinal degeneration, neurosensory hearing loss, and insulin resistance (Stretton et al., 2009).

In addition to the typical C-terminal tubular domain, plant TLPs have a conserved N-terminal F-box domain, which is not present in mammalian TLPs (Gagne et al., 2002; Lai et al., 2004). Previous studies have shown that TLPs have a variety of functions in plants, including growth, development, and disease resistance. TLPs may function in pollen grains, consistent with the fact that *AtTLP6*, *AtTLP7*, and *AtTLP2* are mainly expressed in pollen grains of *Arabidopsis* (Bao et al., 2014). *AtTLP2* is involved in the biosynthesis of homogalacturonic acid in *Arabidopsis* seed coat mucilage (Wang et al., 2019). Fourteen *TLP* genes have been identified in rice (*OsTLPs*), and differential expression analysis confirmed that the members of this group play important roles in processes related to physiological development (Liu, 2008). Expression of each *OsTLP* was induced by infection with *Xanthomonas oryzae* pv. *oryzae*, indicating that the *OsTLP* family is involved in host–pathogen interaction (Kou et al., 2009). *OsTLP2* can bind to the *OsWRKY13* promoter to regulate rice resistance to fungal plague and bacteria (Cai et al., 2008). Tomato *SlTLP1* and *SlTLP2* may have important roles in ethylene-dependent fruit ripening (Zhang et al., 2020); *SlTLFP8* regulates cell size and stomatal density through endoreduplication, reduces water loss, and enhances water use efficiency (Li et al., 2020).

Previous reports have demonstrated the responses of TLPs to various abiotic stresses. During seed germination and seedling growth, *AtTLP3* responds to abiotic stresses such as abscisic acid (ABA), NaCl, and mannitol (North et al., 1997; Bao et al., 2014). *AtTLP9* regulates ABA sensitivity during seed germination and early seedling development (Lai et al., 2004; Chen et al., 2020). Overexpression of *CaTLP1* in chickpeas can enhance tolerance to drought, salt stresses, and ABA (Bhushan et al., 2007). In apples, polyethylene glycol (PEG) treatment up-regulates expression of *MdTLP1*-*MdTLP5* and *MdTLP9* (Xu et al., 2016). Overexpression of apple *MdTLP7* enhances the tolerance of *Arabidopsis* to osmotic, salt, and temperature stresses (Xu et al., 2019). *ZmTLP2* and *ZmTLP11* are significantly up-regulated in maize under drought stress (Chen et al., 2016). *CsTLP8* plays a negative regulatory role in osmotic stress in cucumber, and its effects may be related to ABA (Li S. et al., 2021). Transcriptome analysis has shown that cotton *GhTULPs* are involved in abiotic stresses and tissue development. Overexpression of *GhTULP34* was shown to decrease the germination rate of *Arabidopsis* seeds under salt stress, inhibit root development under osmotic stress, and lead to the closure of plant stomata (Li Z. et al., 2021). In summary, TLPs play key roles in plant growth and development and in responses to biotic and abiotic stresses.

Soybean (*Glycine max*) is one of the most economically important crops in the world, often used as a source of food for humans and livestock because of its rich oil and protein (Papiernik et al., 2005). As global climate change occurs, the adaptability of soybean to its living environment is gradually reduced, causing a demand for stress-tolerant soybean varieties. Further studies are needed to improve soybean tolerance to extreme environments, including various abiotic stresses such as drought and salt (Le et al., 2012). There is little published information about TLPs and their relationship with abiotic stress mechanisms in soybean. In this study, 22 *TLP* genes were identified in the soybean genome, and bioinformatic analyses were conducted to determine their chromosomal locations, gene structures, protein domains, conserved motifs, three-dimensional structures, and *cis*-acting elements. Based on RNA-Seq and quantitative Real-Time Polymerase Chain Reaction (qRT-PCR), we further investigated the role of *GmTLP8* in drought and salt stress responses in soybean, and found that overexpression of *GmTLP8* enhanced tolerance to drought and salt stresses in soybean. These findings provide insights into the function of *GmTLP8*, specifically in abiotic stress responses, and into the importance of GmTLPs more broadly in plant abiotic stress responses.

## Materials and Methods

### Identification of Tubby-Like Proteins in Soybean

Soybean genome, protein, complementary DNA (cDNA) sequences, and gene annotation files were obtained from NCBI^1^ and the Phytozome database^2^ (Finn et al., 2011; Fernandez-Pozo et al., 2015). The Hidden Markov Model (HMM) profile corresponding to the TLP Tub domain (PF01167) from the Pfam protein family database^3^ was used to identify potential TLPs in the soybean genome (*G. max* Wm82. a2.v1) using HMMER v3 (Eddy, 1998; Mistry et al., 2021). Finally, the presence of the Tub domain in each TLP protein sequence was confirmed with the SMART tool^4^ (Letunic et al., 2012) and Pfam database. The molecular weight and isoelectric point data for GmTLPs were calculated by ExPASY^5^ (Artimo et al., 2012). Subcellular localization was predicted with WoLF PSORT^6^.

### Phylogenetic Tree Construction and Multiple Sequence Alignment

The full-length amino acid sequences of TLP members in rice (OsTLPs), *Arabidopsis* (AtTLPs), cotton (GhTLPs), maize (ZmTLPs), apple (MdTLPs), poplar (PtTLPs), wheat (TaTLPs), tomato (SlTLPs), and the newly identified GmTLPs were obtained from NCBI and Phytozome, respectively, and aligned with default parameters using ClustalW (Chenna et al., 2003). An unrooted phylogenetic tree was constructed using the neighbor-joining (NJ) method in MEGAX (version 10.1.8) (Tang et al., 2021) with the following parameters: pairwise deletion; Poisson model; 1000 bootstrap replications.

The amino acid sequence of 22 TLP proteins of soybean aligned using DNAMAN (version 6.0.3).

### Chromosomal Localization, Structural Characterization, and Conserved Motif Analysis

Chromosomal location data for *GmTLP*s were obtained from the Phytozome database. Intron insertion sites were identified by comparing the coding sequence of each *TLP* gene with the corresponding full-length sequence using the Gene Structure Display Server (GSDS) 2.0^7^ (Hu et al., 2015). The conserved domain of the identified soybean GmTLP protein sequences were determined using MEME^8^ with the maximum number of motifs set to 10 (Bailey et al., 2009).

### Protein Domain Analysis and Homology Modeling

Protein sequences of the 22 GmTLPs were submitted to the SMART website^9^ to obtain data related to conserved protein domains, and GSDS 2.0 was used for visual analysis. Three-dimensional models of the Tub domain were built with SWISS-MODEL^10^ (Dong et al., 2019). Tub domain models were obtained for 20 GmTLPs with the protein sequence identity set to ≥30%.

### Expression Patterns of *TLPs* in Soybean

Soybean gene expression files were downloaded from the Soybase website^11^ to analyze the expression patterns of 22 GmTLPs members in different tissues at different developmental stages under normal conditions, including young-leaf, flower, pod, pod shell, seed, root, and nodule. In the database file provided by Soybase website, only 18 members’ tissue differential expression information were found for further analysis. Transcriptome data for GmTLPs members under various abiotic stresses from our previous studies (Wang et al., 2020). 22 GmTLPs members were used for searching in transcriptome data, and their expression levels under normal condition, ABA treatment, drought and salt stresses were analyzed. Finally, the relevant information of 21 members was obtained. TBtools (version 1.075) (Chen et al., 2020) was used for visualization and cluster analysis of *GmTLP* expression patterns.

### Analysis of *Cis*-Acting Elements in *GmTLP* Gene Promoters

*GmTLP* sequences obtained from the Phytozome database were extracted in batches with TBtools, and the 2000 bp upstream promoter sequences of the 22 *GmTLP* genes were obtained and submitted to the online program PlantCARE^12^ to identify *cis*-acting elements. GSDS 2.0 was used for data visualization.

### Plant Materials and Growth Conditions

The soybean variety Zhonghuang39 was used for analysis of *GmTLP* gene expression in this study. Soybeans were grown in 1: 1 vermiculite: humus in a greenhouse with a 16/8 h light/dark cycle, day/night temperatures of 28/20°C, and a relative humidity of 70%. At 14 days, the seedlings at the four-leaf stage were stressed with drought or salt. Referring to previous research methods (Wang et al., 2021), soybean seedlings were removed from soil. For drought stress, the seedlings were placed on filter paper; for salt stress, the seedlings were immersed in 200 mM NaCl solution. The sampling time of drought or salt stress was 0, 0.5, 1, 2, 4, 8, 12, and 24 h. There were three biological replicates per treatment. After treatment, the leaves were frozen in liquid nitrogen and stored at –80°C before further analysis (Xu et al., 2008). These samples were used for qRT-PCR analysis of subsequent *GmTLPs* members.

### RNA Extraction and Quantitative Real-Time Polymerase Chain Reaction

Total RNA was extracted from soybean leaves using a plant RNA extraction kit following the manufacturer’s instructions (TIANGEN, Beijing, China). cDNA was synthesized using the PrimeScript™ RT Reagent Kit (TaKaRa, Shiga, Japan) following the manufacturer’s protocol. Primers (Supplementary Table 2) were designed using Primer Premier 5.0. The soybean *Actin* gene (U60506) was used as the internal control for quantitative real-time PCR (qRT-PCR). There were three technical replicates for each sample. Differential expression was determined from the relative gene expression data using the 2^–Δ^ ^Δ^ *^CT^* method (Le et al., 2011).

### Subcellular Localization of GmTLP8

We constructed an expression vector labeled with green fluorescent protein (hGFP) for subcellular localization analysis. The full-length cDNA sequence of *GmTLP8* was fused to the N-terminal hGFP protein driven by the CaMV35S promoter (Xu et al., 2007). The 35S:GFP vector was used as a control. A PEG4000-mediated method was used to transform the *GmTLP8*-GFP recombinant plasmid into *Arabidopsis* protoplasts (He et al., 2016). After incubation for 18–20 h in the dark at 22°C, the nucleus of *GmTLP8*-GFP protoplasts were specifically stained with 4’, 6-diamidino-2-phenylindole (DAPI). The fluorescence signal was observed using a confocal laser scanning microscope (Zeiss LSM 700, Oberkochen, Germany). There were three technical replicates for each group.

### *Agrobacterium rhizogenes*-Mediated Transformation of Soybean Hairy Roots

The transformation was conducted to produce soybean hairy roots that were characterized by overexpression of *GmTLP8* (*GmTLP8*-OE), RNA interference of *GmTLP8* (*GmTLP8*-RNAi), or with the empty pCAMBIA3301 vector (EV-Control) (Chen et al., 2021). The CDS of *GmTLP8* was amplified without stop codon using gene-specific primer pairs, under the control of the CaMV35S promoter, *GmTLP8* cDNA was ligated into the plant transformation vector pCAMBIA3301 to generate *GmTLP8*-overexpressing (*GmTLP8*-OE) vector. In order to construct the RNAi vector, a 564 bp fragment including the first intron sequence and its reverse complement was synthesized (Biomed, Beijing, China) and inserted into pCAMBIA3301 to generate the pCAMBIA3301-*GmTLP8*-RNAi (*GmTLP8*-RNAi) vector. The recombinant construct and the empty pCAMBIA3301 (EV-Control) vector were transferred into *A. rhizogenes* strain K599, as previously described, then injected into soybean (*G. max cv.* Zhonghuang39) hypocotyl for *A. rhizogenes*-mediated transformation of soybean hairy roots (Wang et al., 2009; Du et al., 2018).

The injected plants were placed in a high-humidity greenhouse until hairy roots were generated at the infected site and had grown to ∼5 cm in length. After cutting off the original tap root 0.5 cm below the infected site, the seedlings were transplanted into fertilized soil and cultivated in a greenhouse at 25°C with a 16/8 h light/dark photoperiod for 7 days (Yu et al., 2021). The qRT-PCR analysis of *GmTLP8* expression in *GmTLP8*-OE, EV-control and *GmTLP8*-RNAi transgenic hairy root plants before processing (Supplementary Figure 2G). Each of the hairy root-related experiments was replicated at least three times independently. The primers of *GmTLP8*-3301-F and *GmTLP8*-3301-R were listed in Supplementary Table 2.

### Drought and Salt Stress Assays of Soybean Hairy Root Composite Plants

Transgenic hairy root composite soybean plants were used in drought and salt stress assays after 7 days of normal growth. For drought treatment, soybean plants were grown for 7 days without watering; for NaCl treatment, soybean plants were treated with 150 mM NaCl for 3 days. Drought and salt treatment experiments were conducted a minimum of three times. Both the treated and untreated soybean hairy roots were washed with water prior to RNA isolation and physiological/biochemical experiments.

### Measurements of Physiological Indexes

Several physiological parameters were measured in transgenic *GmTLP8*-OE, EV-Control, and *GmTLP8*-RNAi lines after the drought and NaCl treatments, namely levels of proline (Pro), malondialdehyde (MDA), hydrogen peroxide (H_2_O_2_), superoxide anion (O_2_^–^), and chlorophyll. Measurements were taken in soybean leaves using appropriate assay kits (Cominbio, Suzhou, China) following the manufacturer’s instructions. All measurements were performed in three biological replicates.

### Leaf Staining With 3,3-Diaminobenzidine and Nitro Blue Tetrazolium

Leaves from the three transgenic lines were stained with 3,3-diaminobenzidine and nitro blue tetrazolium after drought or salt stress treatment. The leaves were immersed in DAB solution or NBT staining solution (Solarbio, Beijing, China) for 18 or 14 h, respectively. Samples were then destained in a boiling solution of 3: 1 anhydrous ethanol: glycerol until the leaves were white (Du et al., 2018). Images were taken using a Canon 50D camera (Canon, Tokyo, Japan). There were three biological replicates for each plant line–treatment group combination.

## Results

### Identification of Tubby-Like Proteins in Soybean Genome

Twenty-two GmTLP family members were identified in this study. The SMART and Pfam databases were used to confirm the presence of the conserved Tub domain in all of the putative TLP proteins. Twenty-two *GmTLP* genes were unevenly distributed across 13 chromosomes of soybean. According to their positions on chromosomes, we named them *GmTLP1* to *GmTLP22*. The details of TLPs in soybean, such as the coding sequence (CDS) length, amino acid length (aa), molecular weight (MW), isoelectric point (p*I*), and subcellular location are shown in Table 1.

**TABLE 1:** Basic information of TLPs in soybean.

Name	Gene ID	CDS(bp)	Chr	Protein(aa)	MW(Da)	p*I*	Subcellular localization
GmTLP1	Glyma.01G173700	1249	1	415	46341.13	9.37	Nucl/cyto
GmTLP2	Glyma.02G055300	1246	2	414	46043.62	9.18	Chlo
GmTLP3	Glyma.02G081800	1276	2	424	47548.48	9.46	Nucl
GmTLP4	Glyma.02G152700	1204	2	400	44763.46	9.47	Nucl
GmTLP5	Glyma.07G147700	553	7	183	20813.28	9.43	Cyto
GmTLP6	Glyma.07G251800	1081	7	359	40220.18	9.06	Cyto
GmTLP7	Glyma.08G183100	1285	8	427	48059.27	9.48	Nucl
GmTLP8	Glyma.10G224900	1294	10	430	48068.12	9.65	Nucl
GmTLP9	Glyma.11G069400	1246	11	414	46094.90	9.36	Cyto
GmTLP10	Glyma.12G115200	889	12	295	33619.95	7.54	Cyto
GmTLP11	Glyma.12G230000	1138	12	378	41678.40	9.37	Mito
GmTLP12	Glyma.13G214900	1171	13	389	43646.37	9.44	Mito
GmTLP13	Glyma.13G269600	1147	13	381	41987.79	9.32	Mito
GmTLP14	Glyma.13G371500	1072	13	356	40004.66	9.63	Nucl
GmTLP15	Glyma.14G073500	1273	14	423	47357.84	9.29	Nucl
GmTLP16	Glyma.15G049500	1285	15	427	47952.01	9.41	Nucl
GmTLP17	Glyma.15G098200	1159	15	385	43331.98	9.30	Nucl
GmTLP18	Glyma.16G138100	1246	16	414	46248.94	9.01	Chlo
GmTLP19	Glyma.16G167200	1276	16	424	47621.54	9.46	Nucl
GmTLP20	Glyma.17G022700	1108	17	392	44015.49	9.07	Nucl
GmTLP21	Glyma.17G251500	1366	17	454	50583.48	9.41	Mito
GmTLP22	Glyma.20G166900	1294	20	430	48203.31	9.61	Nucl

Among the 22 GmTLPs, the protein length ranged from 183 (*GmTLP5*) to 454 amino acids (*GmTLP21*). The minimum protein MW was 20.8 kDa (*GmTLP5*), and the maximum was 50.6 kDa (*GmTLP21*). The p*I* ranged from 7.54 in *GmTLP10* to 9.65 in *GmTLP8*. Twelve of the proteins were predicted to be located in the nucleus, five in the cytosol, four in the mitochondria, and two in the chloroplast (Table 1), with *GmTLP1* predicted to be located in either the nucleus or cytosol.

### Chromosome Distribution, Phylogenetic Analysis, and Multiple Sequence Alignment

**FIGURE 1:**
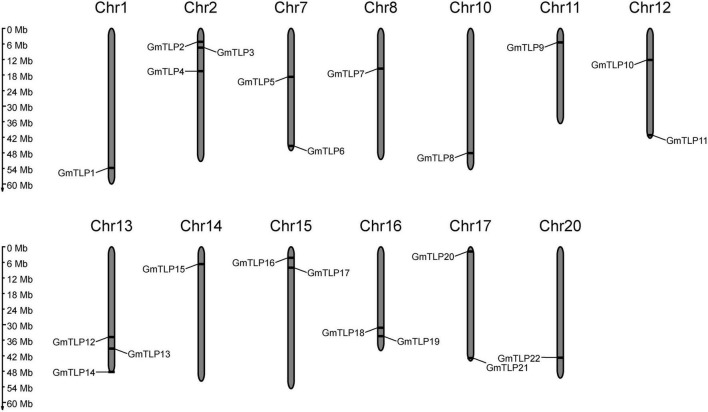
Chromosomal distribution of the 22 putative *TLP* genes identified in soybean. The scale bar at left indicates the size of the chromosomes.

A physical location map of the *GmTLPs* was drawn using physical location data from the soybean genome. The 22 *GmTLP* genes were distributed across 13 chromosomes, which were chromosome 1, 2, 7, 8, 10, 11, 12, 13, 14, 15, 16, 17, 20, respectively. There were three genes on chromosomes 2 and 13, two genes on chromosomes 7, 12, 15, 16, and 17, and only one gene each on chromosomes 1, 8, 10, 11, 14, and 20 (Figure 1).

**FIGURE 2:**
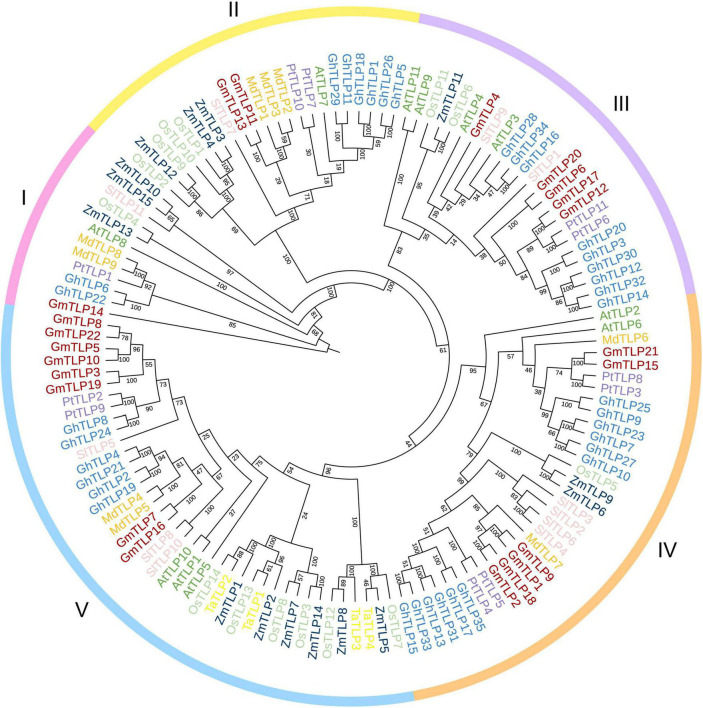
Phylogenetic analysis of TLP proteins. The full-length amino acid sequences of TLP proteins from *Arabidopsis* (AtTLPs), rice (OsTLPs), maize (ZmTLPs), tomato (SlTLPs), apple (MdTLPs), cotton (GhTLPs), poplar (PtTLPs), wheat (TaTLPs), and soybean (GmTLPs) were aligned using ClustalW. The phylogenetic tree was constructed using the NJ (Neighbor-joining) method with 1000 bootstrap replicates. Distinct subfamilies are marked with different colors.

To reveal the phylogenetic relationships between TLPs in different plant species, an unrooted phylogenetic tree was constructed by comparing the amino acid sequences for all of the known TLP members in several species, totaling 132 proteins. There were 11 from *Arabidopsis* (Lai et al., 2004), 15 from maize (Chen et al., 2016), 14 from rice (Liu, 2008), four from wheat (Hong et al., 2015), 11 from tomato (Zhang et al., 2020), nine from apple (Xu et al., 2016), 11 from poplar (Yang et al., 2008), and 35 from cotton (Li Z. et al., 2021) in addition to the 22 putative TLPs identified in soybean. Phylogenetic tree was divided into five groups based on protein homology, and there were one, two, five, six, and eight GmTLP members in groups I, II, III, IV, and V, respectively (Figure 2).

**FIGURE 3:**
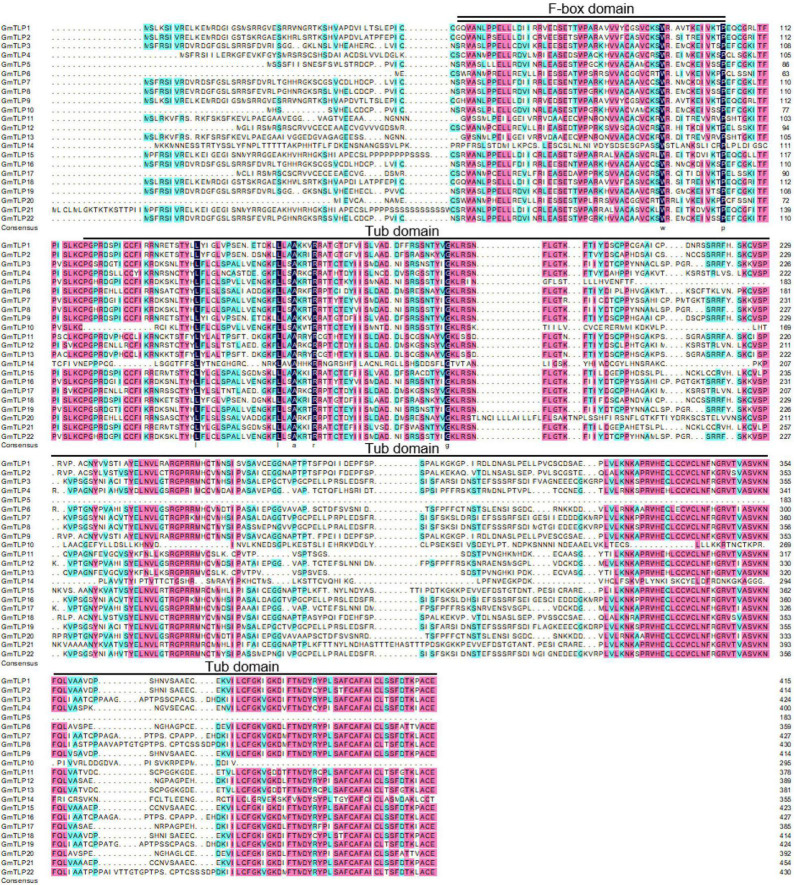
Multiple sequence alignment of GmTLP proteins from soybean using DNAMAN. Black, pink and light blue shading, respectively, represent amino acids with 100, ≥75, and 50% similarity of amino acids. The locations of the F-box domain and tubby domain are indicated with double and single solid lines above the sequences, respectively. The alignment is generated by the ClustalW program.

The results of multiple sequence alignment showed that the positions of F-box domain and Tub domain in GmTLP protein sequence were located in the front and rear segments of the sequence (Figure 3).

### Gene Structure and Motifs in GmTLPs

**FIGURE 4:**
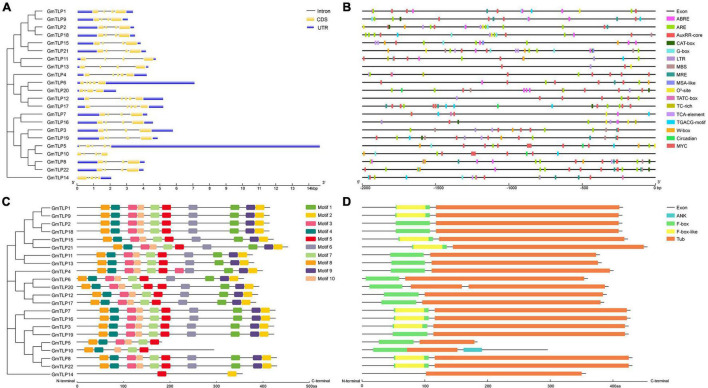
Gene structures, *cis*-acting elements, motifs and conserved domains analysis of GmTLPs. **(A)** Phylogenetic relationships (left) and gene structures (right) of GmTLPs. The phylogenetic tree was constructed using MEGAX; the different classes of TLP proteins make up separate clades. The schematic diagram shows gene structure. Introns and exons are indicated by black lines and yellow boxes, respectively. The lengths of introns and exons in each gene are displayed proportionally. **(B)** Predicted *cis*-acting elements in the *GmTLP* promoters. Distinct color blocks indicate different *cis*-elements, including ABRE, ARE, LTR, TC-rich, MBS, MRE, G-box, TCA-element, TGACG-motif, W-box, circadian, MSA-like, O_2_^–^-site, TATC-box, AuxRR-core, CAT-box, and MYC. The upstream distance from the translation start site can be estimated using the scale at the bottom. **(C)** Phylogenetic relationships (left) and putative motifs (right) of GmTLPs. Conserved motifs were identified using the MEME website and TBtools software. Ten putative motifs are indicated by colored boxes. **(D)** Conserved domain analyses of GmTLPs. The length of each protein can be estimated using the scale at the bottom.

We analyzed the gene structure of the 22 *GmTLPs* using GSDS 2.0 online to determine the intron and exon distribution of each (Figure 4A). A total of 15 genes contained three introns and four exons each, and the other seven genes contained four introns and five exons.

A total of 10 conserved motifs (*E* ≤ 0.01) were analyzed using the MEME website to explore conservation and diversity of soybean TLPs. Among the 22 GmTLP family members, 19 contained all 10 motifs, with GmTLP4 containing two copies of motif 3 and GmTLP20 containing two copies of motif 5. GmTLP5, GmTLP10, and GmTLP14 contained six, five, and two motifs, respectively (Figure 4C). Consensus sequences for putative motifs are shown in Supplementary Figure 1.

### Conserved Domain Analysis and Three-Dimensional Modeling

**FIGURE 5:**
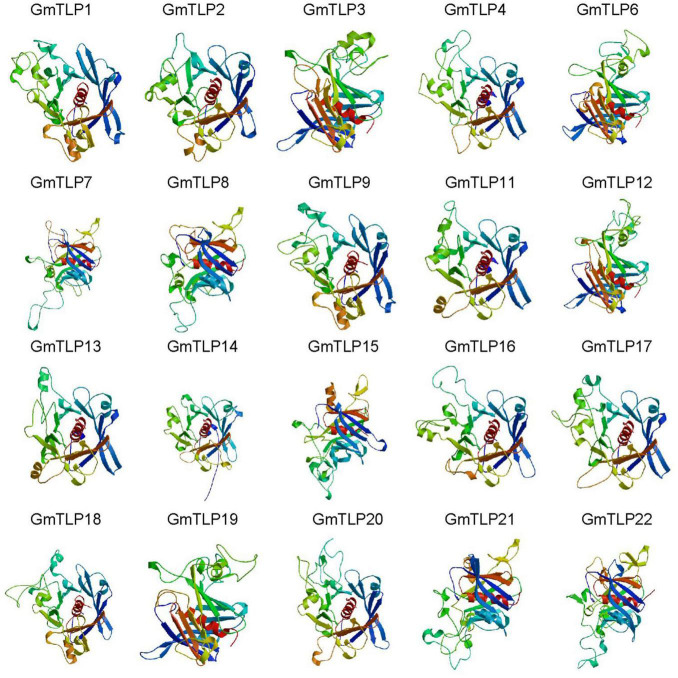
Homology modeling of the 3D structure of GmTLP Tub domains. The α-helices are shown in red, and β-barrels are shown in different colors surround the α-helices. GmTLP, soybean tubby-like protein.

From the Pfam database, we found that two conserved domains in GmTLPs were Tub (PF01167) and F-box (PF00646). We then analyzed the conserved protein domains using both the SMART website (Figure 4D) and homology modeling in SWISS-MODEL (Figure 5).

The results of conserved domain analysis showed that 20 of the GmTLPs contained one F-box domain in the N-terminal region and one Tub domain in the C-terminal region. GmTLP4 had a Tub domain in the C-terminal region but no F-box domain in the N-terminal region, and GmTLP20 contained one F-box domain in the N-terminal region and two Tub domains in the C-terminal region. The analysis also revealed one ANK domain in the C-terminal region of GmTLP10, the function of which was not clear (Figure 4D).

The homology modeling was a useful tool for the prediction of protein structure, and protein structural information was often more valuable than sequence data alone in determining protein function. We generated three-dimensional (3D) models of the Tub domains for 20 of the GmTLPs. These models showed that the Tub domain of each GmTLP was closed by a β-barrel with 12 anti-parallel strands and a central hydrophobic α-helix, which is a typical structure for a Tub domain (Figure 5).

### Promoter Regions of *GmTLPs* Contain Various Stress Response Elements

*Cis-*acting regulatory elements play an important role in modulating gene expression. To understand transcriptional regulation of *GmTLPs*, we identified *cis*-acting elements within the promoter region of each *GmTLP* gene, defined as the 2000 bp region upstream of the start codon (Figure 4B and Supplementary Table 1). Results showed that most *cis*-acting elements in *GmTLP* promoters were involved in hormone or stress responses.

The main hormone-related *cis-*acting elements identified were ABA response element (ABRE), TATC-box (gibberellin), AuxRR core (auxin), TCA element (salicylic acid), and the TGACG motif (methyl jasmonate). Among the *GmTLP* promoters, 14 genes contained ABRE, four contained a TATC-box, six contained the AuxRR core, 11 contained a TCA element, and 12 contained a TGACG motif. This indicated that the *GmTLPs* may be involved in hormone-related responses.

The abiotic stress *cis-*acting elements identified were as follows: anaerobic inducing element (ARE), low temperature response element (LTR), a MYB binding site involved in drought induction (MBS), a drought and salt response element (MYC), and a defense and stress response element (TC-rich element). MYC, which was previously reported to be involved in drought and salt stress-induced responses, was revealed to be distributed in all of the *GmTLP* promoter sequences. In addition, 20 *GmTLP* promoters contained ARE, seven contained LTR, seven contained MBS, and six contained TC-rich elements. The presence of these *cis-*acting elements related to abiotic stresses indicated that *GmTLPs* are abiotic stress-responsive.

Among the 22 *GmTLPs*, 14 contained ABREs, indicating that these genes can be regulated by ABA. Drought and salt response element MYCs were present in all 22 *GmTLP* promoter sequences, strongly suggesting that *GmTLP* members are involved in the responses to those stressors (Zuo et al., 2020). In total, *cis*-acting element analysis indicated that most members of the *GmTLP* family may be regulated by ABA in response to drought and salt stresses.

### Tissue-Specific Expression Patterns of *TLPs* in Soybean

**FIGURE 6:**
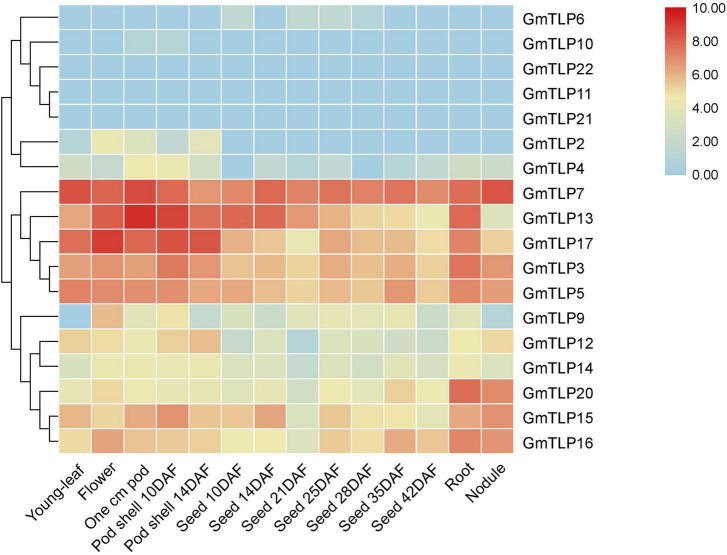
Expression profiles of *GmTLPs* from six soybean tissues. Gene expression was analyzed in soybean young-leaf, flower, pod, pod shell, seed, root, and nodule. The abundance of each transcript (in log_10_-based FPKM) is represented by the color bar. Red indicates higher and blue indicates lower expression levels.

To understand expression patterns of *TLPs* during the growth and development of soybean and throughout different plant tissues, publicly available transcriptome sequencing data from the SoyBase database were analyzed. For 18 *GmTLP* members, we analyzed gene expression levels in different plant tissues including young-leaf, flower, pod, pod shell, seed, root, and nodule. The results showed that *GmTLP7* was extremely high expressed in all seven tissues. *GmTLP6*, *10*, *22*, *11*, and *21* were expressed at extremely low levels or not expressed in seven tissues. *GmTLP2*, *4*, *9*, *12*, and *14* were expressed in some tissues, but not in others. *GmTLP13*, *17*, *3*, *5*, *20*, *15*, and *16* were expressed in all seven tissues, with extremely high expression in some tissues and extremely low expression in others. Phylogenetic analysis divided the 18 *GmTLP* members into different groups, and members within each group shared similarities at the expression level in all seven tissues (Figure 6). The results showed a great deal of spatiotemporal difference in *GmTLP* expression levels.

### Expression Pattern Analysis of *GmTLPs* Under Abiotic Stresses

**FIGURE 7:**
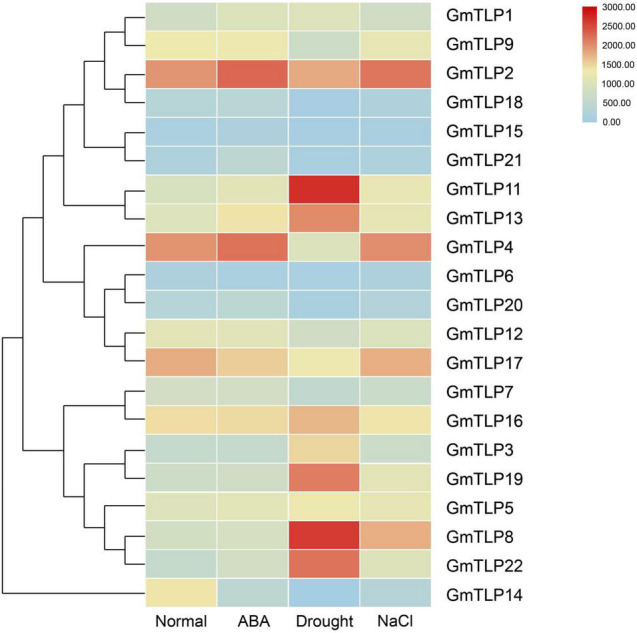
Expression profiles of *GmTLPs* under normal condition (Normal), ABA treatment (ABA), drought (Drought) and salt (NaCl) stresses. The expression abundance of each transcript (in log_10_-based FPKM) is represented by the color. Red indicates higher and blue indicates lower expression levels.

We used a previously published transcriptome sequencing database to quantify the expression of *GmTLPs* under normal condition, ABA treatment, drought and salt stresses (Wang et al., 2020), and screened 21 *GmTLP* members (Figure 7). The results showed that *GmTLP21* was up-regulated and *GmTLP14* was down-regulated under ABA treatment. Under drought stress, six genes were up-regulated, and *GmTLP3*, *8*, *11*, *13*, *19*, and *22*, four genes were down-regulated, and *GmTLP14*, *15*, *18*, and *20*, respectively; Under salt stress, *GmTLP8* was up-regulated and *GmTLP14* was down-regulated. The *P*-value of data in abiotic stress expression profiles is shown in Supplementary Table 3.

### Responses of *GmTLP8* to Various Treatments

**FIGURE 8:**
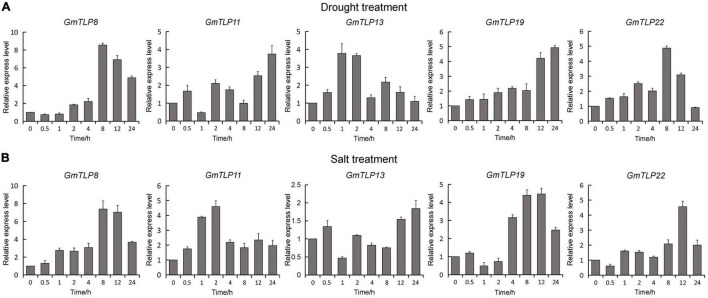
Expression patterns of *GmTLPs* under drought and salt treatments. *GmTLP* gene expression was measured in response to drought **(A)** and NaCl **(B)** treatments using qRT-PCR. The *x*-axes show the duration of treatment and *y*-axes depict relative expression level. The data are shown as mean ± standard deviation (SD) of three technical and three biological replicates.

According to the expression profiles of GmTLPs under different abiotic stresses, five genes (*GmTLP8*, *11*, *13*, *19*, and *22*) up-regulated expression under drought stress and one gene (*GmTLP8*) under salt stress were selected for qRT-PCR analysis to further verify their relative expression levels under drought and NaCl treatments. The selected genes were *GmTLP8*, *11*, *13*, *19*, and *22*. Under drought treatment (Figure 8A), *GmTLP8* expression peaked at 8 h (with an 8.5-fold increase compared to 0 h), *GmTLP11* at 24 h (3.7-fold), *GmTLP13* at 1 h (3.7-fold), *GmTLP19* at 24 h (4.9-fold), and *GmTLP22* at 8 h (4.8-fold). Under salt treatment (Figure 8B), *GmTLP8* expression peaked at 8 h (7.3-fold), *GmTLP11* at 2 h (4.5-fold), *GmTLP13* at 24 h (1.8-fold), *GmTLP19* at 12 h (4.4-fold), and *GmTLP22* at 12 h (4.5-fold). These results showed that *GmTLP8* was the most highly expressed in response to drought and salt treatments, and it was therefore selected for further study.

### Subcellular Localization

**FIGURE 9:**
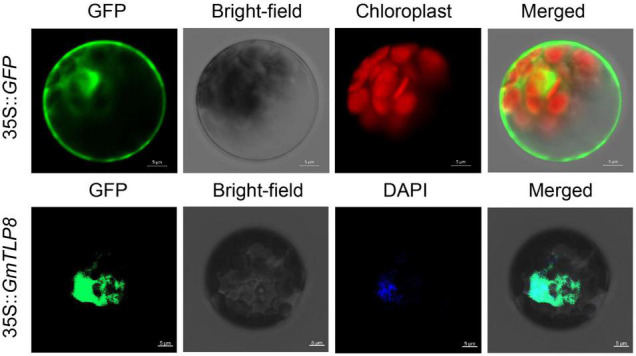
Subcellular localization of *GmTLP8*-hGFP fusion protein. 35S:GFP was used as the control. Scale bar = 5 μm.

To determine the subcellular localization of GmTLP8, the open reading frame (ORF) sequence (excluding the termination codon of *GmTLP8*) was fused with the N-terminal of the humanized green fluorescent protein (hGFP) reporter and co-transformed into *Arabidopsis* protoplasts. A 35S:hGFP as the control, the fluorescence signal in the cells was detected by confocal laser scanning microscopy. The fluorescence of GmTLP8 was detected in the nucleus and cytoplasm, while the fluorescence of the control 35S:hGFP was observed in the whole cell. DAPI staining also showed the localization of GmTLP8 in the nucleus (Figure 9). It suggests that GmTLP8 act as a transcription factor in the nucleus (Li S. et al., 2021).

### *GmTLP8* Improved Drought and Salt Tolerance in Soybean Transformants

**FIGURE 10:**
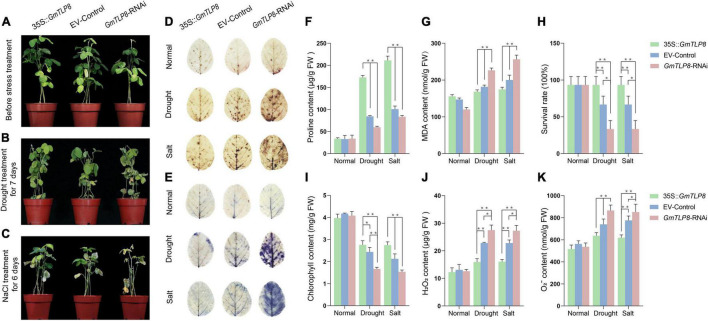
Analysis of the function of soybean *GmTLP8*. **(A–C)** Phenotypes of transgenic soybean hairy root composites *GmTLP8*-OE (35S:*GmTLP8*), EV-Control (empty plasmid), and *GmTLP8*-RNAi plants under **(A)** normal conditions, **(B)** drought stress, or **(C)** salt stress. **(D)** DAB and **(E)** NBT leaf staining of the *GmTLP8-*OE, EV-Control, and *GmTLP8*-RNAi lines under normal conditions and drought or salt stress. The depth of color corresponds to the concentrations of H_2_O_2_ and O_2_^–^ in the leaves. **(F)** Proline (Pro) content, **(G)** malondialdehyde (MDA) content, **(H)** survival rate, **(I)** chlorophyll content, **(J)** H_2_O_2_ content, and **(K)** O_2_^–^ content in transgenic soybean hairy root composite plants and EV-control plants under normal conditions and drought or salt stress. Vertical bars indicate ± SD of three technical and three biological replicates. *p < 0.05, ^**^p < 0.01 (Student’ s *t*-test).

The stress-tolerant effect of *GmTLP8* in soybean was explored using transgenic soybean hairy root composite plants. The hairy roots of *GmTLP8*-OE, EV-Control and *GmTLP8*-RNAi transgenic lines were used to analyze the relative expression level of *GmTLP8*. qRT-PCR analysis showed that the expression level of *GmTLP8*-OE transgenic hairy roots was significantly higher than that in EV-Control, and the expression level of *GmTLP8*-RNAi transgenic hairy roots was lower than that in EV-Control (Supplementary Figure 2G). Under normal growth conditions, no significant differences were observed between *GmTLP8*-OE, the EV-Control, and *GmTLP8*-RNAi lines (Figure 10A). However, after exposure to drought (Figure 10B) and salt (Figure 10C) treatments, there were significant phenotypic differences between *GmTLP8*-OE, EV-Control, and *GmTLP8*-RNAi plants. Compared with EV-Control, *GmTLP8*-RNAi plants showed more severe leaf dehydration and wilting stress phenotype, whereas *GmTLP8*-OE showed fewer rolled leaves and a delayed leaf wilting phenotype. The survival rates of *GmTLP8*-OE, EV-Control, and *GmTLP8*-RNAi lines under drought stress were 93, 67, and 40%, respectively; these survival rates were comparable to those of salt-stressed plants (Figure 10H).

Proline (Pro), malondialdehyde (MDA), hydrogen peroxide (H_2_O_2_), and superoxide anion (O_2_^–^) levels are important indicators of the effects of abiotic stresses on plant growth (Leng et al., 2021). Proline is a protective agent against osmotic stress; MDA reflects the degree of lipid oxidative damage; H_2_O_2_ and O_2_^–^ play immune and signal transduction roles, although excessive accumulation may lead to cell membrane damage (Du et al., 2018; Zhang et al., 2019). Chlorophyll levels are an important indicator of plant photosynthetic capacity (Tanaka and Tanaka, 2006). To further analyze the potential physiological mechanism of *GmTLP8* in plant stress tolerance, we measured the levels of Pro, MDA, H_2_O_2_, O_2_^–^, and chlorophyll in the leaves of *GmTLP8*-OE, EV-Control, and *GmTLP8*-RNAi plants under normal growth conditions and under drought or salt stress (Figures 10F,G,I–K). Levels of Pro and chlorophyll were higher in *GmTLP8*-OE compared with EV-Control, whereas levels of MDA, H_2_O_2_, and O_2_^–^ were lower. In contrast, the *GmTLP8*-RNAi lines had lower Pro and chlorophyll levels but higher MDA, H_2_O_2_, and O_2_^–^ levels than EV-Control.

H_2_O_2_ and O_2_^–^, produced by the reactive oxygen species (ROS) pathway in leaf cells under abiotic stress, were measured to assess the degree of damage in leaf cells (Cui et al., 2019). This was done using DAB and NBT to stain the leaves of *GmTLP8*-OE, EV-Control, and *GmTLP8*-RNAi plants (Figures 10D,E). Under normal conditions, leaves from the *GmTLP8*-OE, EV-Control, and *GmTLP8*-RNAi lines showed minimal staining, with no significant difference between lines. Under drought and salt stresses, compared with EV-Control, *GmTLP8*-OE leaves showed shallow staining, whereas *GmTLP8*-RNAi showed deeper staining. These results demonstrated that the *GmTLP8*-OE line had lower levels of leaf damage and the *GmTLP8*-RNAi line had more severe leaf damage compared to EV-Control in response to exogenous abiotic stresses. The results of staining leaves with DAB and NBT were consistent with the physiological indexes of H_2_O_2_ and O_2_^–^ contents.

### *GmTLP8* Activated Stress-Responsive Genes in Soybean

**FIGURE 11:**
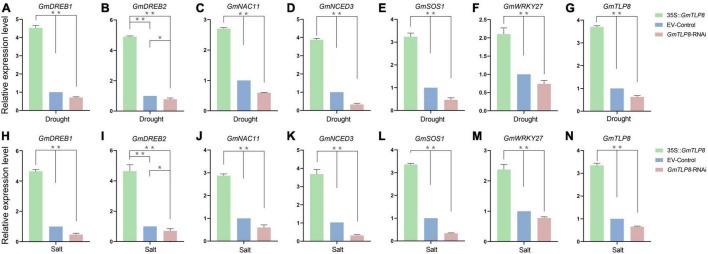
*GmTLP8* regulates stress-responsive gene expression in transgenic soybean plants. **(A–F)** Expression levels of selected stress-related genes in transgenic soybean plants under drought stress. **(H–M)** Expression levels of selected stress-related genes in transgenic soybean plants under salt stress. **(G)** Expression levels of *GmTLP8* in transgenic soybean plants under drought stress. **(N)** Expression levels of *GmTLP8* in transgenic soybean plants under salt stress. Vertical bars indicate ± SD of three technical and three biological replicates. *p < 0.05, ^**^p < 0.01 (Student’ s *t*-test).

To analyze the potential stress tolerance mechanism of *GmTLP8*, genes known to be involved in drought and salt stress responses were selected, namely *GmDREB1* (Kasuga et al., 1999), *GmDREB2* (Chen et al., 2007), *GmNAC11* (An et al., 2018), *GmNCED3* (Pandey and Gautam, 2020), *GmSOS1* (Ma et al., 2020), and *GmWRKY27* (Wang et al., 2015) (Figures 11A–F,H–M). Expression of these genes in the hairy roots of *GmTLP8*-OE, EV-Control, and *GmTLP8*-RNAi transgenic soybean lines were measured via qRT-PCR. Plants were drought-treated by withholding water for 7 days or salt-treated with 150 mM NaCl for 3 days. Under normal growth conditions, the selected stress responsive genes were expressed at lower levels in all three plant lines compared to plants that had been exposed to drought or salt stress (Supplementary Figures 2A–F and Figures 11A–F,H–M). In plants that had been stressed, compared with EV-Control, the six stress-related genes were significantly up-regulated in *GmTLP8*-OE plants and down-regulated in *GmTLP8*-RNAi plants. These results suggest that overexpression of *GmTLP8* may activate expression of downstream drought- and salt-response genes.

## Discussion

Previous reports have proven that TLP family members participate in plant growth and development, response to abiotic stress, and can also be involved in the ABA signaling pathway (North et al., 1997; Lai et al., 2004; Bao et al., 2014; Chen et al., 2020). Also, reports have confirmed the resistance of TLPs members in *Arabidopsis* (Lai et al., 2004), maize (Chen et al., 2016), wheat (Hong et al., 2015), tomato (Zhang et al., 2020), apple (Xu et al., 2016), and cotton (Li Z. et al., 2021) to abiotic stress, but no report has been found in soybean. We used the NJ method to construct the phylogenetic tree of multiple species. According to the homology of protein sequences, they were divided into five groups, which were similar to the phylogenetic tree group in cotton previously reported (Li Z. et al., 2021). In group I, there is only one GmTLPs member, GmTLP14, which is the same as AtTLP8 in *Arabidopsis* previously reported (Lai et al., 2004). N-terminal of GmTLP14 and AtTLP8 do not contain F-Box domain, indicating that they may come from the same ancestor, so they are classified as the same group (Figure 2).

Gene structure analysis showed that each member of the *GmTLPs* had introns and exons, and their numbers were similar to those previously reported in *Arabidopsis* (Lai et al., 2004), indicating that the soybean *TLPs* was evolutionary conserved (Figure 4A). Motif analysis showed that except GmTLP14 containing two motifs, the number of motifs contained by other members was not less than five (Figure 4C). Analysis of protein conserved domains showed that except GmTLP14 had only one domain, other members contained two/three conserved domains (Figure 4D). Multiple sequence alignment marks the protein sites of two key conserved domains (Figure 3), consistent with the domain distribution shown in Figure 4D. Above results showed that the protein structures of other members of GmTLPs were similar except GmTLP14. Further analysis of transcriptome data showed that the up-regulated gene was *GmTLP8* under drought and salt stresses, while the down-regulated gene was *GmTLP14* (Figure 7). The down-regulated expression of *GmTLP14* under drought and salt stresses might be due to the lack of N-terminal F-box domain (Figure 4D).

Two key conserved domains are in plant TLPs, the F-box at the N-terminal and the Tub domain at the C-terminal, and these differ from the conserved domains in mammalian TLPs. In mammals, TLPs are binary transcription factors; the N-terminal induces transcriptional activation, and the Tub domain binds to double-stranded DNA (Boggon et al., 1999; Li S. et al., 2021). In plants, the N-terminal F-box can participate in the formation of the Skp1-Cullin1-F-box (SCF) complex, which is an important part of E3 ubiquitin ligase and can participate in protein ubiquitination process (Gagne et al., 2002). It has been reported that GhTULP34 interacts with the subunit GhSKP1A of the SCF complex to form a functional SCF-type E3 ligase, which may be involved in the response of plants to abiotic stresses (Li Z. et al., 2021). *Arabidopsis* AtTLPs and wheat TaTULPs have been shown to interact with specific S-phase kinase-associated protein 1 (SKP1)-like proteins (Lai et al., 2004; Bao et al., 2014; Hong et al., 2015). These findings suggest that TLPs may play a role as subunits of the SCF complex in plants. Yeast two-hybrid assays showed that AtTLP7 and AtTLP11 interacted with NDR1/HIN1-like protein NHL6 (Song et al., 2019). Because both AtTLP11 and AtTLP7 are functional E3 ligases (Bao et al., 2014), it is possible that AtTLP11 and AtTLP7 redundantly manipulate the function of NHL6 by regulating its protein turnover (Bao et al., 2016). The above reports confirmed that the TLPs family, as F-box proteins, played a key role in protein ubiquitination, and may play a key role in plant response to various adverse environmental conditions. Based on these findings, it is speculated that GmTLP14 may be due to the lack of F-Box domain that affects the protein ubiquitination process and then down-regulates its expression under drought and salt stresses. However, the detailed functions of *GmTLP14* gene need to be verified by related experiments. In this study, through RNA-Seq transcriptome data analysis and qRT-PCR verification, we determined the up-regulated expression of *GmTLP8* under drought and salt stresses for subsequent studies (Figures 7, 8). Conserved domain analysis showed that GmTLP8 had two key conserved domains, namely, F-Box and Tub domains (Figure 4D), 3D modeling showed the integrity of GmTLP8 C-terminal tubby structure, which might play a role in the response of its to abiotic stress (Figure 5).

In this study, *Agrobacterium rhizogenes*-mediated transformation of soybean hairy roots was used to induce transgenic roots in soybean to study the function of *GmTLP8* gene (Kereszt et al., 2007). Through phenotypic observation, leaf staining and physiological index analysis of soybean, it was confirmed that the overexpression of *GmTLP8* enhanced the tolerance of soybean to drought and salt stresses (Figure 10). However, this genetic transformation mode is transient expression and cannot be stably inherited to the next generation through sexual reproduction. Therefore, further exploration of the application of *GmTLP8* gene in transgenic drought-resistant and salt-resistant soybean needs further research on transformation.

Previous studies identified genes that play important roles in response to drought and salt stresses. To further analyze the molecular mechanism of *GmTLP8* in regulating stress tolerance, we chose several confirmed stress-related genes (Figure 11 and Supplementary Figure 2). *GmDREB1*, *GmDREB2*, *GmNAC11*, and *GmWRKY27* can specifically recognize and bind to *cis*-acting elements to up-regulate the expression of downstream stress-responsive genes, improving stress tolerance (Marè et al., 2004; Tran et al., 2004; Xu et al., 2011; Bouaziz et al., 2013, 2015; Sarkar et al., 2019). *GmNCED3* is considered to be an important contributor to ABA synthesis and its overexpression enhances drought tolerance in seedlings (Li et al., 2019). *GmSOS1* improves the salt tolerance of plants, potentially playing a role in Na^+^ extrusion out of the roots and regulation of Na^+^ transport from roots to shoots (Nie et al., 2015; Cao et al., 2018). These selected stress-related genes were up-regulated in *GmTLP8*-OE plants under drought and salt treatments. Taken together, these indicated that *GmTLP8* responds to drought and salt stresses by activating stress-related transcription factors and the SOS pathway, which provides a scientific basis for further analysis of the function of *GmTLP8* gene under drought and salt stresses. However, further studies were needed to fully elucidate its internal mechanism in abiotic stress response.

## Conclusion

In the present study, we identified 22 *TLP* genes in the soybean genome. Based on expression patterns in response to abiotic stresses, we found that GmTLP14 showed different structural characteristics and expression patterns from most other members, but the function of *GmTLP14* still needs further experimental verification. In this study, we selected *GmTLP8* with complete structure and up-regulated expression under drought and salt stresses, and verified its expression level under abiotic stress by qRT-PCR. *GmTLP8* was responsive to drought and salt stresses. Overexpression of *GmTLP8* enhanced the tolerance of soybean to drought and salt stresses by activating downstream stress-responsive genes. These results improve understanding of the GmTLP family and provide a basis for further study of the molecular mechanism of *GmTLP8* in soybean abiotic stress responses.

## Data Availability Statement

The datasets presented in this study can be found in online repositories. The names of the repository/repositories and accession number(s) can be found below: https://www.ncbi.nlm.nih.gov/, PRJNA694374.

## Author Contributions

Z-SX coordinated the project, conceived and designed the experiments, and edited the manuscript. H-RX performed the experiments and wrote the first draft. W-LW and Z-SX revised the manuscript. YL, Z-HH, JC, Y-BZ, and MC contributed to data analysis and managed reagents. T-FY, J-DF, J-CZ, and Y-ZM contributed with valuable discussions. All authors reviewed and approved the final manuscript.

## Conflict of Interest

The authors declare that the research was conducted in the absence of any commercial or financial relationships that could be construed as a potential conflict of interest.

## Publisher’s Note

All claims expressed in this article are solely those of the authors and do not necessarily represent those of their affiliated organizations, or those of the publisher, the editors and the reviewers. Any product that may be evaluated in this article, or claim that may be made by its manufacturer, is not guaranteed or endorsed by the publisher.

## Footnotes

1
http://www.ncbi.nlm.nih.gov/

2
https://phytozome.jgi.doe.gov/pz/portal.html

3
http://pfam.xfam.org/

4
http://smart.embl-heidelberg.de/

5
http://web.expasy.org/

6
https://wolfpsort.hgc.jp/

7
http://gsds.cbi.pku.edu.cn

8
https://meme-suite.org/meme/

9
http://smart.embl-heidelberg.de/smart/batch.pl

10
https://swissmodel.expasy.org

11
http://www.SoyBase.org

12
http://bioinformatics.psb.ugent.be/webtools/plantcare/html/

## Abbreviations

TLP: tubby-like protein
ABA: abscisic acid
GFP: green fluorescent protein
MDA: malondialdehyde
PRO: proline
PEG: polyethylene glycol
DAB, 3: 3-diaminobenzidine
NBT: nitro blue tetrazolium
qRT-PCR: quantitative real-time PCR
ABRE: ABA-responsive element
MYC: drought and salt-responsive element
NCED: 9-*cis*-epoxycarotenoid dioxygenase
SCF-complex: Skp1-Cullin1-F-box complex
SKP1-like proteins: S-phase kinase-associated protein 1 like proteins
NHL: NDR1/HIN1-like gene.
