# Supplementary material for: Comprehensive Profiling of Tubby-Like Proteins in Soybean and Roles of the GmTLP8 Gene in Abiotic Stress Responses
Source: Front Plant Sci. 2022 Apr 25;13:844545. doi: 10.3389/fpls.2022.844545 (PMC9083326; doi:10.3389/fpls.2022.844545)
Supplement: Supplementary file 4 [file Image_1.PDF]

Motif	Symbol	Motif Consensus
1		NPSAPEQEKVILQFGKVGKDJFTMDYRYPLSAFQAFAICLSSFDTKLACE
2		LVLKNKAPRWHEQLQCWCLNFRGRVTVASVKNFQLVAATEP
3		QSRWANLPPELLRDVIRRLEASETTWPARKHVVACAAVCKSWREITKEIV
4		KFLLAAKRTRRTTCTEYIISLDADBISRSSNTYVGKLRSNF
5		PEFCGKITFPISLKQPGPRDSPIQCFIKRBKSTSTYYLFLGLSPALLVEN
6		KQVSPKVPAGNYNVAQVSYELNVLGTRGPRRMHCVMHSIPASAIZPGGVA
7		LGTKFTIYDSQPPHSGAKLSP
8		PGQPELLPRSLEDSFRSISFSKSIDNSTEFSSSRFSDIFEAGIEDEEGKV
9		SIVRELKEVRDGIGSLSRRGFEVRLWGGH
10		TPTSFPQIFDEPFSPSPALKGKGPIRDLDNASLPE

Supplementary Figure 1. Consensus sequences for putative motifs.
