# Supplementary material for: Comprehensive Profiling of Tubby-Like Proteins in Soybean and Roles of the GmTLP8 Gene in Abiotic Stress Responses
Source: Front Plant Sci. 2022 Apr 25;13:844545. doi: 10.3389/fpls.2022.844545 (PMC9083326; doi:10.3389/fpls.2022.844545)
Supplement: Supplementary file 5 [file Image_2.PDF]

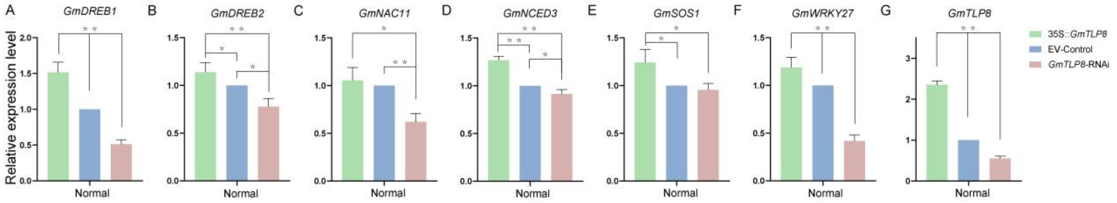

Supplementary Figure 2. Stress response gene expression in *GmTLP8* transgenic soybean hairy root complex plants under normal growth conditions. (A-F) Expression levels of selected stress-related genes in transgenic soybean plants under normal conditions. (G) Expression levels of *GmTLP8* in transgenic soybean plants under normal conditions. Vertical bars indicate ± SD of three technical and three biological replicates. $^{*}p < 0.05$, $^{**}p < 0.01$ (Student’ s *t*-test).
